# Comparison of a Flow Assay for Brucellosis Antibodies with the Reference cELISA Test in West African *Bos indicus*

**DOI:** 10.1371/journal.pone.0005221

**Published:** 2009-04-20

**Authors:** Barend M. deC. Bronsvoort, Bronwyn Koterwas, Fiona Land, Ian G. Handel, James Tucker, Kenton L. Morgan, Vincent N. Tanya, Theresia H. Abdoel, Henk L. Smits

**Affiliations:** 1 Centre for Tropical Veterinary Medicine, The Roslin Institute and Royal (Dick) School of Veterinary Studies, University of Edinburgh, Easter Bush Veterinary Centre, Roslin, Midlothian, United Kingdom; 2 KIT Biomedical Research, Royal Tropical Institute / Koninklijk Instituut voor de Tropen (KIT), Amsterdam, The Netherlands; 3 Department of Bacterial Diseases, FAO/WHO Collaborating Centre for Reference and Research on Brucellosis, Veterinary Laboratories Agency, New Haw, Weybridge, Surrey, United Kingdom; 4 Department Veterinary Clinical Sciences, University of Liverpool, Leahurst Veterinary Teaching Hospital, Neston, Wirral, United Kingdom; 5 Ministry of Scientific Research and Innovation, Yaoundé, Cameroon; University of Liverpool, United Kingdom

## Abstract

Brucellosis is considered by the Food and Agricultural Organisation and the World Health Organisation as one of the most widespread zoonoses in the world. It is a major veterinary public health challenge as animals are almost exclusively the source of infection for people. It is often undiagnosed in both human patients and the animal sources and it is widely acknowledged that the epidemiology of brucellosis in humans and animals is poorly understood, particularly in sub-Saharan Africa. It is therefore important to develop better diagnostic tools in order to improve our understanding of the epidemiology and also for use in the field for disease control and eradication. As with any new diagnostic test, it is essential that it is validated in as many populations as possible in order to characterise its performance and improve the interpretation of its results. This paper describes a comparison between a new lateral flow assasy (LFA) for bovine brucellosis and the widely used cELISA in a no gold standard analysis to estimate test performance in this West African cattle population. A Bayesian formulation of the Hui-Walter latent class model incorporated previous studies' data on sensitivity and specificity of the cELISA. The results indicate that the new LFA is very sensitive (∼87%) and highly specific (∼97%). The analysis also suggests that the current cut-off of the cELSIA may not be optimal for this cattle population but alternative cut-offs did not significantly change the estimates of the LFA. This study demonstrates the potential usefulness of this simple to use test in field based surveillance and control which could be easily adopted for use in developing countries with only basic laboratory facilities.

## Introduction

Brucellosis is considered by the Food and Agricultural Organisation and the World Health Organisation as one of the most widespread zoonosis in the world. Brucellosis in humans (mainly due to *Brucella melitensis* and *B.* abortus) produces an acute febrile disease that may progress to a chronic form. Brucellosis in animals is a sub-acute or chronic disease affecting a range of domestic and wildlife species [1]. Mortality rates may be around 5% higher in calves from seropositive cows [2], with high morbidity rates in adults. Brucellosis it is the leading cause of contagious abortion in livestock. The most important species are *B. abortus*, *B melitensis* and *B. suis* causing abortions, premature births and retained placentae in livestock [3]. Risk factors for human cases often include consumption of fresh dairy products that have not been pasteurized, contact with infected animals or abortive material, handling animals products [4]. Animals are almost exclusively the source of infection for people and therefore any attempts at reducing the human disease burden is dependent on identifying the infected animal source.

Sub-Saharan Africa (SSA) has the world's fastest growing population where livestock rearing is still the principal economic activity supporting livelihoods in the desert, arid grasslands and savannahs where the harsh environment is unsuitable for other forms of agriculture. This disease is considered the most important veterinary public health issue in the region and reducing the burden of infection in livestock through testing and removal of seropositive individual animals should reduce the risk of new human cases, thereby having a major public health impact. However, funding for control programmes has declined in the last 20 years [1] while at the same time there is increasing small-holder dairying and increasing cattle densities in the region. Schelling et al. [5] have estimated the human prevalence of seropositivity in neighbouring Chad to be 3.8%, highlighting the enormous public health issue that is largely being ignored.

The Royal Tropical Institute in the Netherlands has developed a rapid and simple point-of-care test, the *Brucella* IgM/IgG immunochromatographic lateral flow assay for the serodiagnosis of human brucellosis [6], [7]. This has recently been adapted for testing cattle sera [8] and is hereafter referred to as the lateral flow assay (LFA). The LFA is a simplified ELISA for the qualitative detection of antigen specific antibodies in serum or whole blood samples. The assay is based on the binding of specific antibodies to antigen immobilized on a test strip. Bound antibodies are visualized using a secondary antibody conjugated to colloidal gold particles. Binding of the conjugate at the test zone of the assay device is visible by the unaided eye and results may be read within 10 minutes. Application of the assay requires neither specific expertise, equipment or electricity, and tests may be kept in stock without the need for refrigeration.

This paper describes a comparison between this new LFA for bovine brucellosis and the O.I.E. reference cELISA in a no gold standard analysis. The test's sensitivity and specificity were estimated for a West African *Bos indicus* cattle population and the implication of using the test are discussed.

## Methods

### Study Population

The cattle were sampled in the year 2000 as part of a population based study and the design has been described previously [9]. In brief, the study area was the five administrative Divisions, Vina, Mbere, Djerem, Mayo Banyo and Faro Deo of the 64,000 km^2^ Adamawa Province of Cameroon. A two-stage random sampling of herds was carried out based on a sample frame of the 13,006 herds registered for rinderpest vaccination in the Province. Within selected herds 5 juvenile animals (6–24 months old) and 5 adult animals (>24 months old) were randomly selected. Jugular blood samples were collected into 10 ml plain vacutainers which were then centrifuged at 1,100 g for 10 minutes in the field using a ‘Mobilespin’ 12V field centrifuge (Vulcon Technologies) or a hand crank centrifuge (OFI Testing Equipment, Inc.). Approximately 3.5 ml of serum was aliquoted into 2×1.8 ml cryovials (Nunc). The sera were stored at 4°C in a portable gas fridge until they could be frozen and stored at −20°C. The sera were carried to the UK on dry ice and stored at −20°C at the FMDV World Reference Laboratory, Pirbright, UK. The herds were presumed to be unvaccinated because no government licenses have been issued to import vaccines into the country and Brucella was not one of the routine vaccines used by the government veterinary services at the time of the sample collection. The cELISA was performed at the Institute for Animal Health (IAH), Pirbright by staff from the Veterinary Laboratory Agency (VLA), Weybridge in 2003 and the LFA was carried out in 2007. Each testing round was done blind using the same serum samples at IAH.

### cELISA

The cELISA for *Brucella* diagnostic kits is based on detection of the lipopolysaccharide (LPS) antigen of smooth *Brucella* strains. The immunodominant epitope of the LPS is the O-chain which is a homopolymer of 1,2-linked *N*-acylated 4-amino-4, 6-dideoxy-α-D-mannopyranosyl residues [10]. The cELISA was provided and performed by VLA staff according to the O.I.E. Manual of Standards for Diagnostic Tests and Vaccines [11] using the 16 M Melitensis strain as antigen and OPD as the chromogen, stopped with Citric acid. The optical density (OD) was read at 450 nm and the percentage OD of the conjugate (% OD) were calculated as the average OD of the paired sample wells divided by the average OD of the four conjugate wells on the plate. The cELISA used a monoclonal antibody specific to the O-chain polysaccharide portion of the *Bucella* LPS [12]. The standard %OD cut-off of 70% was used initially for interpretation of results but 60% and 50% cut-offs were also explored in the latent class analysis.

### Brucella Lateral Flow Assay (LFA)

The *Brucella* LFA device for the serodiagnosis of bovine brucellosis consists of a porous nitrocellulose detection strip flanked at one end by a reagent pad and at the other end by an absorption pad. A sample application pad flanks the reagent pad in turn. The composite strip is contained in a plastic assay device with a round sample well positioned above the sample application pad and a test result window positioned above the detection zone of the strip. The detection zone contains two distinct lines, a test line and a control line. The test line was manufactured by spraying *Brucella*-LPS antigen onto the nitrocellulose strip and the control line by spraying bovine immunoglobulin G (IgG) antibodies. Test and control lines were sprayed using a BioDot Quanti 2000 BioJet. As *Brucella*-specific antigen a LPS extract from a solid culture of *Brucella abortus* strain 1119-3 was used [6]. A detection reagent was prepared by conjugating affinity purified antibodies against cattle Ig (H+L) antibodies to 40 nm colloidal gold particles and this conjugates were sprayed onto the conjugate pad of the composite strip using the AirJet of the BioDot Quanti 2000 machine. Tests are performed by the addition of 5 µl serum to the sample pad of the assay device followed by the addition of 130 µl sterile running fluid consisting of phosphate-buffered saline, pH 7.6, containing 1.67% bovine serum albumin and 3% Tween 20. Test results were read after 10 min by visual inspection for staining of the antigen and control lines. Tests were scored negative when no staining was observed at the test line and scored positive when the test line stains. The control line should stain in all cases. The test line may stain at different intensities, and positive results may be subjectively rated 1+ when staining is weak, 2+ when staining is moderately strong, 3+ when staining is strong, and 4+ when staining is very strong. Devices sealed in a humidity resistant foil and containing silica may be stored at 4–27°C without loss of activity. The stain of exposed tests is stable after drying.

### Statistical Analysis

Hui & Walter [13] introduced a latent class approach to the evaluation of diagnostic tests in absence of a “gold-standard”. The Hui-Walter paradigm requires two (or more) tests evaluated in two (or more) populations. This model assumes that: (i) the prevalence of the disease is different within each population; (ii) the tests have the same properties across populations; (iii) and the tests are conditionally independent given the disease status.

The Bayesian version of the Hui-Walter model [14] assumes that for the *i*th subpopulation the counts (**O_i_**) of the different combinations of test results, +/+, +/−, −/+ and −/− for the two tests, follow a multinomial distribution:
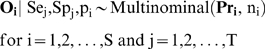
where S is the number of subpopulations, T is the number of tests and **Pr_i_** is a vector of probabilities of observing the individual combinations of test results. Conditioning on the (latent) disease status, these probabilities can be specified using the Se_j_ and Sp_j_ of the tests and the prevalence (p_i_) of the subpopulations. As an example, for two tests the probability of observing both tests positive in the *i*th subpopulation is given as:
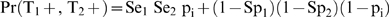
The other three probabilities for the three test scenarios may be similarly derived.

In a Bayesian analysis all parameters are expressed as random variables. Hence, prior distributions for the test properties and the prevalence within the subpopulations must be specified. For those parameters where no information was available the distributions were modelled using uninformative, uniform priors on the interval between zero and one:
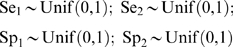



However, there have been a number of recent publications where estimates were given for the Se and Sp of the cELISA. Table 1 gives the references and the sample sizes of the studies involved. These were then incorporated into the Bayesian framework as priors for the Se and Sp of the cELISA (see appendix for coding of full model) to inform posterior estimates.

**Table 1:** Estimates of Se and Sp and sample sizes used from literature search.

Paper	Cut-off	Se	Sp	N Se	R Se	N Sp	R Sp
Fosgate et al. (2003)[36]	30 PI	0.839	0.926	63	53	323	299
Gall et al. (1998) [37]	26 PI	0.975	0.983	1857	1810	2613	2569
McGiven et al. (2003) [28]	70 %OD	0.952	0.997	146	139	1440	1436
Neilson et al. (1995) [38]	30 PI	1	0.997	636	636	1446	1442
Nielson et al. (1996) [39]	Not given	0.986	0.977	654	645	1508	1473
Stack et al (1999) [12]	60 %OD	0.979	1	147	144	640	640

Se = sensitivity; Sp = specificity; N Se = number in sample for Se estimation; R Se = number test positive; N Sp = number in sample for Sp estimation; R Sp = number test negative; PI = percentage inhibition; %OD = percentage of the OD of the conjugate.

If the two tests can not be reasonably assumed to be independent then the Hui and Walter model must be extended to account for the covariance structure between the two tests [15], [16] as below:
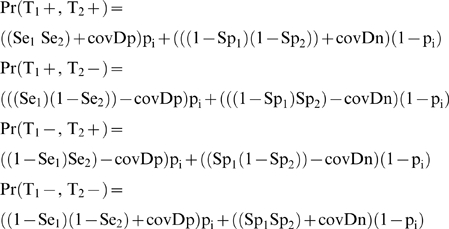



The covDp and the covDn are the covariances between the two tests when the animal is diseased and when it is not diseased respectively. The covariance between the test outcomes for infected subpopulations satisfies (Se_1_−1)(1−Se_2_)≤covDp≤(min[Se_1_,Se_2_]−(Se_1_Se_2_)) and for the non-infected subpopulation, (Sp_1_−1)(1−Sp_2_)≤covDp≤(min[Sp_1_,Sp_2_]−(Sp_1_Sp_2_)) [17]. Therefore, for instance, a uniform ((Se_1_−1)(1−Se_2_), (min[Se_1_,Se_2_]−(Se_1_Se_2_))) prior distribution can be used for covDp.

The model was implemented in WinBUGS [18]. For this analysis, the first 200,000 iterations were discarded as a burn-in and the following 300,000 iterations were kept for posterior inference. Convergence of the chain after the initial burn-in was assessed by visual inspection of the time-series plots for the parameters as well as Gelman-Rubin diagnostic plots using three sample chains with dispersed starting values [19]. The models were compared using the Deviance Information Criteria (DIC) [20].

The positive predicted values (PPV) were calculated for a number of test parameter combinations over a range of lower potential seroprevalences using the following formula:
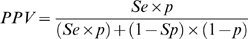
Where Se is the estimated test sensitivity, Sp is the estimated test specificity, p is the true seroprevalence in the population.

## Results

**Figure 1:**
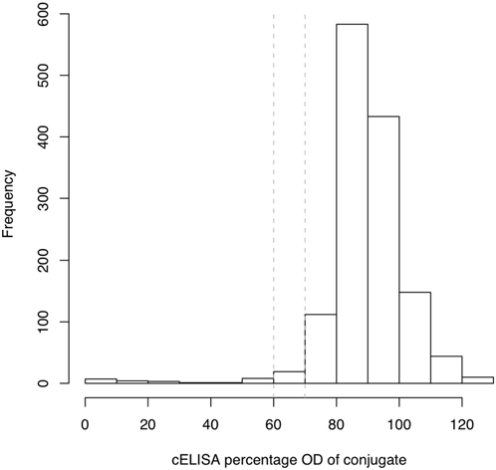
Histogram of cELISA percentage OD of the conjugate. Histogram of the distribution of OD values expressed as the percentage OD of the conjugate for 1,375 cattle from Adamawa Province Cameroon. The standard cut-off of <70% OD and the <60% OD for a positive test result are also plotted as grey vertical lines.

A total of 1,375 serum samples from 146 herds from the Adamawa Province of Cameroon were screened with both tests. The %OD for the cELISA shown in Figure 1 clearly demonstrates a bimodal distribution of the values across the sampled population. The plot suggests that there may be a better %OD cut-off of 50% for the cELISA in this African cattle population which we have explored later in the analysis.

### Model 1: Informative cELISA priors with conditional dependence and a %OD cut-off of 70%

**Figure 2:**
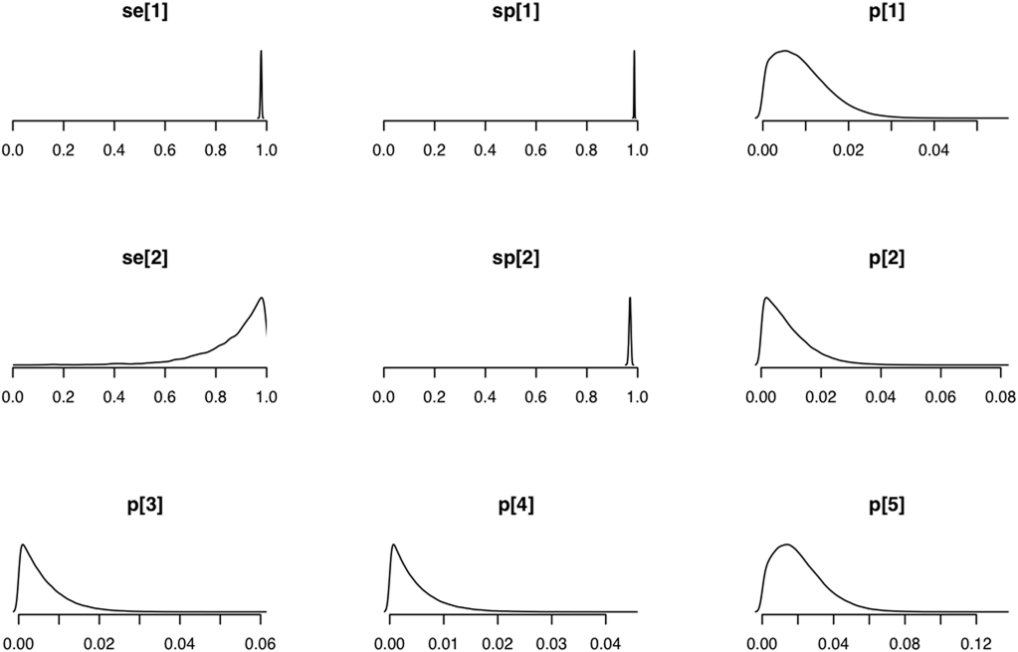
Posterior distributions of test parameters for the LFA from model 1. Posterior distributions from Model 1 using prior estimates for the cELISA from literature and conditional dependence between tests at a %OD cut-off of 70% for the cELISA. Se[1] = Se of the cELISA; Se[2] = Se of the LFA; Sp[1] = Sp of the cELISA; Sp[2] = Sp of the LFA; p[1] = p for Vina; p[2] = p for Mbere; p[3] = p for Djerem; p[4] = p for Mayo Banyo; p[5] = p for Faro et Deo.

**Figure 3:**
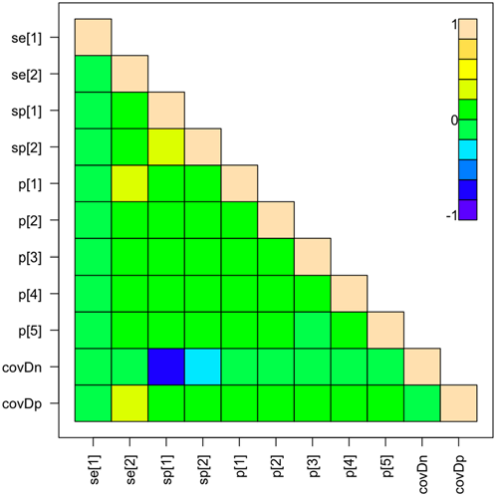
Cross correlation plot of parameters from model 1. Cross correlation plot of parameters from Model 1 using prior estimates for the cELISA from literature and conditional dependence between tests at a %OD cut-off of 70% for the cELISA. Se[1] = Se of the cELISA; Se[2] = Se of the LFA; Sp[1] = Sp of the cELISA; Sp[2] = Sp of the LFA; p[1] = p for Vina; p[2] = p for Mbere; p[3] = p for Djerem; p[4] = p for Mayo Banyo; p[5] = p for Faro et Deo.

The first model used the standard %OD of 70% as the cut-off and all the prior information available from previous studies of the cELISA (Table 1) while the LFA was given non-informative beta(1,1) priors. The Se, Sp, and p estimates from the model are given in Table 2 and the full posterior distributions plotted in Figure 2. The distributions show that the prior estimates of Se and Sp for the cELISA, taken from previous studies, dominate the information from the data. The estimate of the Se of the LFA from this model has a mean of 0.869 with a very high Sp of 0.970. The model showed good mixing and convergence a DIC of 53.95. The animal-level prevalences were all very low at <0.01 for four of the five administrative Divisions and 0.02 in the highest in Faro et Deo. The cross correlations between the parameters in model 1 are plotted in Figure 3. This shows that the Se_LFA_ is moderately correlated with the covDp and the Sp of both tests is quite strongly negatively correlated with the covDn.

**Table 2:** Parameter estimates for Model 1 with 95% Bayesian credibility interval (BCI) using prior estimates for the cELISA from literature and conditional dependence between tests at a 70% OD cut-off for the cELISA. DIC for model 1 was 53.95.

Parameter	Mean	2.5% BCI	97.5% BCI
Se (LFA)	0.869	0.503	0.996
Sp(LFA)	0.970	0.962	0.977
Se(cELSIA)	0.978	0.973	0.983
Sp(cELISA)	0.987	0.984	0.989
covDn	0.012	0.009	0.015
covDp	0.008	−0.005	0.020
P(Vina)	0.009	0.001	0.024
P(Mbere)	0.009	0.000	0.028
P(Djerem)	0.006	0.000	0.021
P(Mayo Banyo)	0.005	0.000	0.016
P(Faro et Deo)	0.021	0.001	0.055

### Model 2 Informative priors with %OD of 70% as cut-off and assuming conditional independence

If the assumption of conditional independence is used (i.e. covariances are fixed at zero) the parameter estimates change only slightly but the DIC for this model increased to 82.02 suggesting a much poorer model fit and a lower predictive ability, so this model was not considered further.

### Model 3 Conditional dependence with %OD of 60% and 50% as the cut-off for the cELSIA with non-informative priors

From Figure 1 the cut-off for the cELISA could reasonably be adjusted to 60% or 50% OD. However, there is little or no information on how the tests perform at these cut-offs and so we attempted to fit a model using non-informative priors Unif(0,1) for both tests' Se and Sp and allowing conditional dependence. However, in spite of being a large population sample, there was insufficient information in the data to estimate all 11 parameters in the model with any certainty. The model was explored further by adjusting the priors for the cELISA as it was argued that even with the shift in cut-off it was reasonable to believe the Se_cELISA_ and Sp_cELSIA_ to be high, although probably the Se_cELISA_ might have dropped. Beta(5,2) and Beta(2,1) priors were tried for the cELISA and LFA parameters respectively but the credible intervals were still very large because there was insufficient information from the data to identify the tests' parameters due to the low prevalence of disease. For example at 50% OD cut-off the 95% BCI for the Se_LFA_ ranged from 0.008 to 0.926 and the 95% BCI for the p_[1]_ from 0.004 to 0.931. In order to try and improve the certainty of the estimates we simplified the model and dropped the conditional dependence component and therefore 2 parameters but in spite of convergence of the chains the credible intervals were still extremely large. These were not explored any further as there was no further information available to set priors for the cELSIA at these cut-offs.

**Figure 4:**
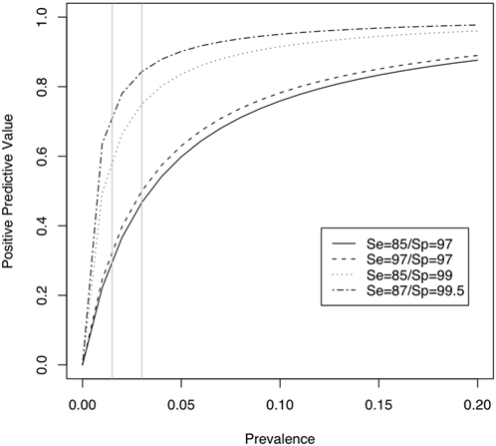
The positive predictive value of the LFA. The PPV of combinations of test Se and Sp over a range of prevalences of brucellosis. The gray vertical lines mark the 1.5 and 3% prevalence levels.

In figure 4 the PPV of a positive test result for the LFA is plotted against the prevalence of infection for various combinations of test Se and Sp, and the PPV for the LFA at each of these combinations for the observed prevalence of brucellosis in the cattle population in Adamawa Province is indicated.

## Discussion

An important component of any disease control effort is the ability to identify infected or infectious animals and treat or remove them from the population. In the case of brucellosis, identifying infected animals and removing from the herd is key to the control of the disease in both the livestock and the human populations. Although some diagnostic or screening tests are referred to as the “gold standard”, there are in fact few perfect diagnostic tests and there is always a compromise between performance and cost. For example, many screening programmes use tests with less than perfect Sp if this gives a high Se and then use a more specific test to confirm any positive animals.

The best estimates using all the information available suggest that the LFA is a sensitive and highly specific test. This means that at the low seroprevalence of 1–2% found in this region of West Africa, the PPV of a positive test result for the LFA (assuming a Se of ∼87% and a Sp of ∼97%) is approximately 50%. However, the PPV markedly increases as the Sp increases or if the prevalence of disease is higher. The high Sp of the LFA ensures the prognostic value of a positive LFA test results is still very good when testing animals in areas where the prevalence of brucellosis is relatively low. Its ease of use makes it a very attractive screening tool for use in bovines and removes the need for laboratory facilities and plate readers.

Model 1 gives very low seroprevalence estimates for cattle in the Adamawa in 2000. The seroprevalence appears to be between 0.5% (95% BCI: 0–1.6%) in Mayo Banyo and 2.1% (95% BCI: 0.1–5.5%) in Faro et Deo. This contrasts with previous estimates in the Lake Chad region where in the 1980s much higher seroprevalences of ∼30% were reported for Cameroon and 19.6% for the Adamawa Province [21]. They are more in line with more recent estimates of between 5 and 10% reported from an abattoir survey in Western Province Cameroon in 2003 [22] and 7% in neighbouring Chad in 2000 [5] although these were different populations owned by different ethnic groups with different husbandry approaches. However, it is not the focus of this paper to enter a detailed discussion of brucellosis in Cameroon, rather to reflect on the utility of this new test.

The principal methods of diagnosis of bovine brucellosis are culture, the complement fixation test, serum agglutination test, Rose-Bengal test (RBT), indirect enzyme-linked immunosorbent assay and more recently the competitive ELISA (cELISA) and the fluorescent polarisation assay. However, identification of the organism is complex and time consuming and not suitable for large scale screening exercises. Guidelines set by the O.I.E. describe methods and diagnostic thresholds for each of these tests [23]. In the African setting the rose Bengal test (RBT) is widely used for its simplicity and field suitability, for example in Ethiopia and Uganda [24], [25]. The sensitivity and specificity of these tests in the field in Africa is not well described. Recent studies of the various tests has suggested that the RBT may have a Se between 80 and 90% and a Sp around 86% [26], [27]. Estimates for the Se of the cELISA are in the high 90s while the Sp appear to range more widely from 60–99.7% [26]–[28]. Though this study did not compare the LFA directly with the RBT, our results suggest that the LFA should be as sensitive as the RBT but have a much higher specificity. This has the advantage that, except in areas with very low prevalence, the PPV will be very good. The LFA is probably not ideal for large scale screening but could provide a very useful tool in transition countries to identify animals in small holder herds that are infected so that they can be removed or their milk rejected or for providing public health advice to farmers following abortions in their herds.

Most brucellosis serological tests depend on the detection of antibodies to smooth *Brucella* LPS (SLPS). These tests are typically developed using *B. abortus* SLPS given the focus on its importance in the serodiagnosis of humans. Even so, different *Brucella* species with the same LPS form will cross react as it is very similar [29]. *B. melitensis* and *B. suis* contain SLPS while *B. ovis* and *B. canis* have rough LPS [30]. There has been some suggestion [31]–[33] that there can be problems with cross-reactivity to *Yersinia pestis* where it is present but there was no way to explore this here. LFAs have also been developed for testing other livestock species for brucellosis and the use of these LFAs could be equally attractive [8]. In spite of this limitation all the tests based on this antigen have proved useful in the past and this should not detract from the potential of this new test with its higher Se and Sp. The serum used here was originally collected in 2000 and was tested for Brucella antibodies in 2003 and 2007 requiring thawing and freezing. There is not a large literature on the effects of freezing and thaw on antibodies but reports in both the veterinary and human medical literature suggest that they are very robust and that there is no evidence for a significant decline in antibody levels in samples following repeated freeze/thaw cycles [34], [35]

In conclusion, in the absence of a gold standard, the Bayesian formulation of the Hui and Walter model provides the most reliable estimates of diagnostic Se and Sp as well as an unbiased estimate of true prevalence (though this was not the particular focus of this analysis). Using the comparison with the cELISA at a cut-off of 70% and adjusting for conditional dependence and prior estimates of Se and Sp for the cELISA gives the best model and estimates of 87% (95% BCI: 50.3–99.6%) Se and 97% (95% BCI: 96.2–97.7) Sp for the LFA reflecting the much higher certainty in the Sp estimates than those of Se. The distribution of %OD values suggests that a lower cut-off of 60% or even 50% might be appropriate for the cELISA. Although this was explored, without good estimates of the cELSIA Se and Sp at these cut-offs, it was not possible to get estimates with useful credibility intervals for the Se and Sp of the LFA. These results support previous studies and suggest that the LFA may provide a very useful public health tool for control of brucellosis.

Good quality data on the impact of brucellosis in sub-Saharan Africa is still lacking. Brucellosis in cattle is prevalent and widespread in sub-Saharan Africa and the disease is endemic in most pastoral systems. Because culling and other methods to control brucellosis are not widely used in sub-Saharan Africa, long-term chronic infections could be common and these provide a steady supply of infectious organisms. While animals with chronic disease may be difficult to detect by serology, the presence of seropositive animals provide a good indication of ongoing transmission. Further studies looking for presence of brucellosis in humans, confirming the presence of brucellosis in livestock by culture and correlating seropositivity in herds with disease manifestations such as abortions are required to determine the impact of the disease [1].
